# Neuro-inflammatory effects of photodegradative products of bilirubin

**DOI:** 10.1038/s41598-018-25684-2

**Published:** 2018-05-10

**Authors:** J. Jašprová, M Dal Ben, D. Hurný, S. Hwang, K. Žížalová, J. Kotek, R. J. Wong, D. K. Stevenson, S. Gazzin, C. Tiribelli, L. Vítek

**Affiliations:** 1 Institute of Medical Biochemistry and Laboratory Diagnostics, 1st Faculty of Medicine, Charles University, Prague, Czech Republic; 2 Fondazione Italiana Fegato – ONLUS, AREA Science Park Basovizza, Trieste, Italy; 3 Department of Inorganic Chemistry and Department of Organic Chemistry, Faculty of Science, Charles University, Prague, Czech Republic; 4 Department of Chemical and Systems Biology, Stanford University School of Medicine, Stanford, CA USA; 5 Department of Pediatrics, Stanford University School of Medicine, Stanford, CA USA; 6 4th Department of Internal Medicine, 1st Faculty of Medicine, Charles University, Prague, Czech Republic; 7 Department of Medical, Surgical, and Health Sciences, University of Trieste, Trieste, Italy

## Abstract

Phototherapy was introduced in the early 1950’s, and is the primary treatment of severe neonatal jaundice or Crigler-Najjar syndrome. Nevertheless, the potential biological effects of the products generated from the photodegradation of bilirubin during phototherapy remain unknown. This is very relevant in light of recent clinical observations demonstrating that the use of aggressive phototherapy can increase morbidity or even mortality, in extremely low birthweight (ELBW) infants. The aim of our study was to investigate the effects of bilirubin, lumirubin (LR, its major photo-oxidative product), and BOX A and B (its monopyrrolic oxidative products) on the central nervous system (CNS) using *in vitro* and *ex vivo* experimental models. The effects of bilirubin photoproducts on cell viability and expression of selected genes were tested in human fibroblasts, three human CNS cell lines (neuroblastoma SH-SY5Y, microglial HMC3, and glioblastoma U-87 cell lines), and organotypic rat hippocampal slices. Neither bilirubin nor its photo-oxidative products affected cell viability in any of our models. In contrast, LR in biologically-relevant concentrations (25 μM) significantly increased gene expression of several pro-inflammatory genes as well as production of TNF-α in organotypic rat hippocampal slices. These findings might underlie the adverse outcomes observed in ELBW infants undergoing aggressive phototherapy.

## Introduction

Since its discovery in the early 1950’s^1^, phototherapy (PT) has been used as the primary treatment of neonatal hyperbilirubinemia. Possible adverse effects of PT include the bronze baby syndrome, water loss, damage to unprotected eyes^2^, and/or hypocalcemia^3^. Quite surprisingly, recent studies suggest that PT might also be associated with increased risk of ileus^4^, allergic diseases^5^, type 1 diabetes^6^, cancer^7–9^, and even mortality^10,11^, especially in extremely low birthweight (ELBW) neonates.

**Figure 1:**
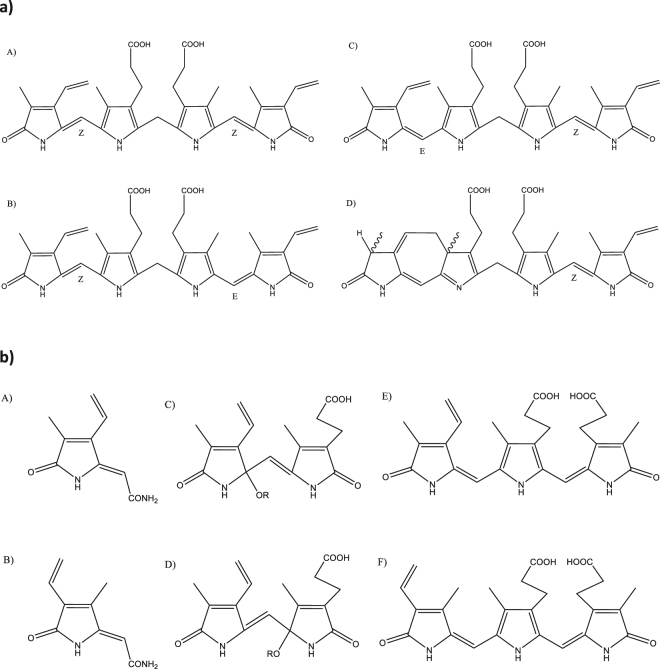
(**a**) Bilirubin and bilirubin PIs. (A) *Z,Z*-Bilirubin IXα; (B) *Z,E*-Bilirubin IXα; (C) *E,Z*-Bilirubin IXα; (D) *Z*-Lumirubin Ixα. (**b**) Bilirubin oxidation products. Monopyrrolic: (A) BOX A, (B) BOX B; Dipyrrolic: (C,D) Propentdyopents; Tripyrrolic: (E) Biopyrrin a, (F) Biopyrrin b.

Photochemical reactions of bilirubin occurring during light exposure lead to the formation of more polar bilirubin photoderivatives, thereby enhancing the elimination of bilirubin elimination^12,13^. Although these photodegradative products are generally regarded as being benign^2^, potential undesirable biological effects have never been properly investigated. During PT, unconjugated bilirubin (UCB) is converted into photoisomers (PIs), *Z,E-* and *E,Z*-bilirubins and lumirubin (LR) (Fig. 1a)^14^; and photo-oxidative products (monopyrrolic, dipyrrolic, and/or tripyrrolic) (Fig. 1b)^15,16^. Whether the PT light itself, bilirubin, or the photo-products formed from bilirubin during PT may cause adverse effects remains unclear.

To this end, we investigated the effects of UCB, LR (its major photoproduct), and the monopyrrolic bilirubin oxidative products, BOX A and BOX B, on cell viability as well as on the expression of several pro-inflammatory and oxidative stress-related genes by using *in vitro* and *ex vivo* experimental models.

## Results

### The effect of bilirubin photo-oxidative products on cell viability

We recently demonstrated that short-term exposures of a mixture of bilirubin PIs to human neuroblastoma SH-SY5Y cells did not affect cell viability, but modulated the expression of genes involved in cell cycle regulation^17^. In this current study, we extended these preliminary findings by investigating the effects of UCB, LR, BOX A, and BOX B exposures on the viability of fibroblasts, several neuronal cell types, and organotypic rat hippocampal slices.

To assess the recently reported biological impact of BOXes^18^, we first assessed cell viability of normal human skin fibroblasts and glucose-6-phosphate dehydrogenase (G6PD)-deficient fibroblasts (known to be sensitive to oxidative stress^19^) after exposure to various concentrations of BOX A or BOX B for 4 h to mimic the early phase of PT. We found that cell viability was not affected in either normal or G6PD-deficient fibroblast (Table 1) even at BOX concentrations far exceeding physiologic levels (up to 200 μmol/L).

**Table 1:** Viability of human skin fibroblasts and G6PD-deficient fibroblasts exposed to BOX A and BOX B for 4 h.

µmol/L	Normal Fibroblasts		G6PD-Deficient Fibroblasts	
	BOX A	BOX B	BOX A	BOX B
10	94.1 ± 6.4%	91.8 ± 3.8%	92.7 ± 2.2%	105.2 ± 4.1%
50	89.9 ± 4.3%	86.5 ± 5.7%	101.2 ± 10.0%	101.8 ± 4.7%
100	90.1 ± 2.9%	86.8 ± 1.4%	101.8 ± 6.9%	101.7 ± 4.2%
200	90.0 ± 5.2%	88.8 ± 3.3%	104.6 ± 8.8%	98.8 ± 1.5%

Based on these findings, we then investigated the effects of bilirubin photo-oxidative products on cell viability *in vitro* using three central nervous system (CNS) models: (1) SH-SY5Y cells, a human neuroblastoma line, which has been used for studying neuronal metabolism; (2) U-87 cells, a human glioblastoma line, which resemble astrocytes; and (3) HMC3 cells, a human microglial line, which are resident CNS macrophages. For all cell lines, exposures to UCB, LR, BOX A, or BOX B for 4 h did not affect cell viability even at the relatively high concentration used (25 μmol/L) (Table 2).

**Table 2:** Viability of human CNS cell lines exposed to UCB, LR, BOX A and BOX B for 4 h.

Cell line	Ctrl	UCB	LR	BOX A	BOX B
SH-SY5Y	100 ± 2.2%	93.0 ± 3.4%	109.3 ± 9.8%	108.9 ± 9.7%	105.3 ± 9.0%
HMC3	100 ± 0.8%	90.5 ± 5.8%*	104.0 ± 4.0%	101.3 ± 6.5%	101.2 ± 3.6%
U-87	100 ± 1.0%	103.8 ± 10.6%	119.6 ± 9.7%*	114.5 ± 15.1%	107.4 ± 12.4%

Cell viability was evaluated by MTT assay. Concentration of tested compounds = 25 μmol/L. Ctrl, control; UCB, unconjugated bilirubin; LR, lumirubin.

*p < 0.05 vs. Ctrl.

**Figure 2:**
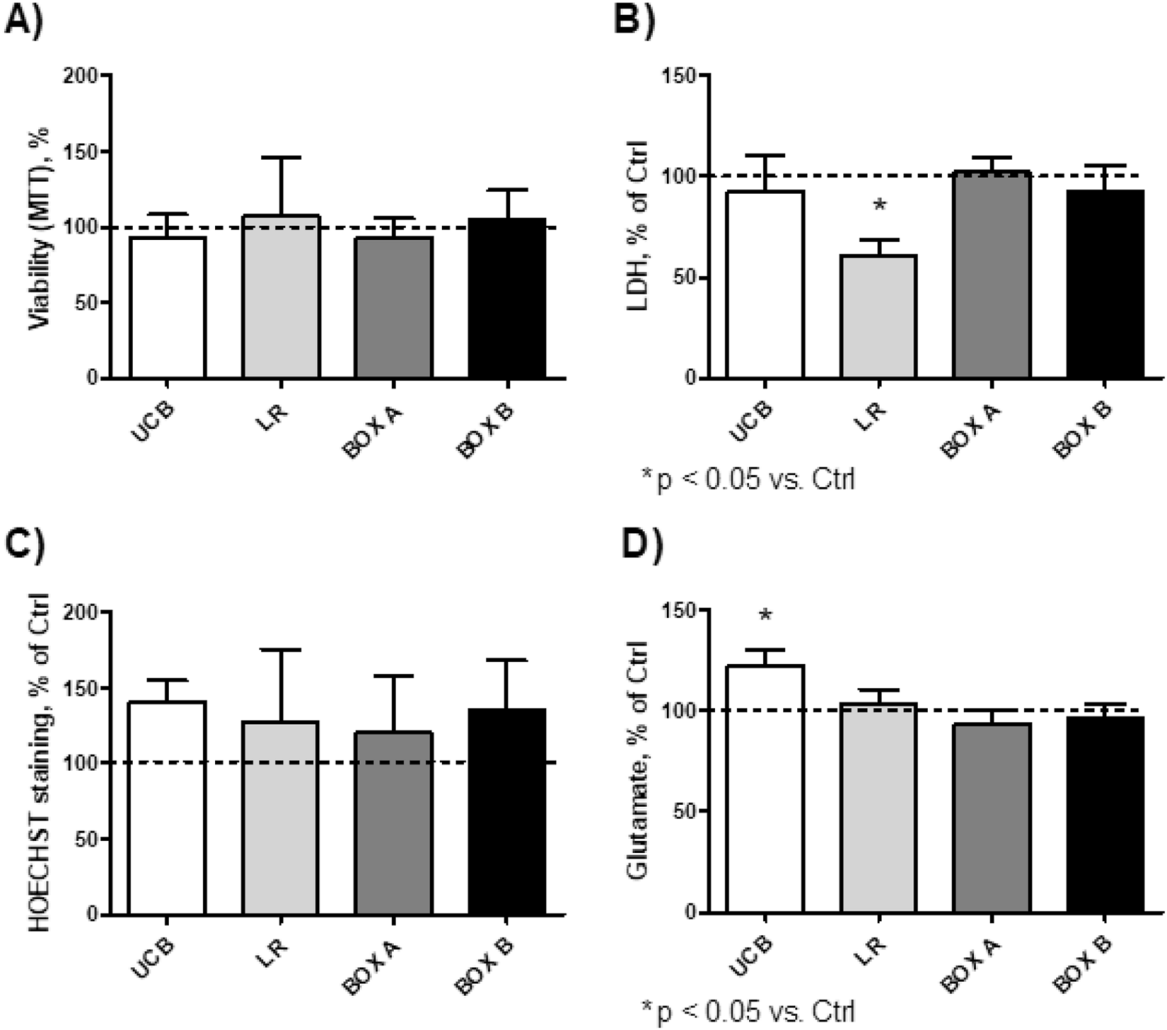
Viability of organotypic rat hippocampal slices after 4 h of exposure to UCB, LR, BOX A, or BOX B. Cell viability was evaluated by MTT assay (**A**), LDH release (**B**), HOECHST staining (**C**), and glutamate release (**D**). The values acquired for control cells were used to define 100% level. Concentrations of tested compounds = 25 μmol/L.

To see if these *in vitro* effects on individual cell lines correlate *in vivo*, we then performed similar exposure studies using organotypic rat hippocampal slices, which is more representative of the complex physiologic multicellular environment since it preserves intercellular interactions within a maintained extracellular matrix^20^. This model has been used to study the pathogenesis of neurodegenerative diseases and of potential neurotoxic effects by various compounds^21^. Similar to our *in vitro* studies, the viability of organotypic rat hippocampal slices was unchanged compared with controls after 4-h exposures to UCB, LR, BOX A, or BOX B using an MTT assay (Fig. 2A). In addition, no difference in lactate dehydrogenase (LDH) release (a marker of cell damage or death) was observed (Fig. 2B). Furthermore, cell apoptosis, measured by Hoechst staining^22^, was also not significantly different (Fig. 2C). After exposure to UCB, the increase in glutamate release (a marker of excitotoxicity, Fig. 2D) was marginal; whereas, no effects were observed after exposure to LR, BOX A, or BOX B (Fig. 2D). Collectively, all viability studies demonstrated that a short-term exposure to UCB and its photo-oxidative products did not lead to cell damage or apoptosis.

### The effect of bilirubin photo-oxidative products on expression of heme oxygenase-1

**Figure 3:**
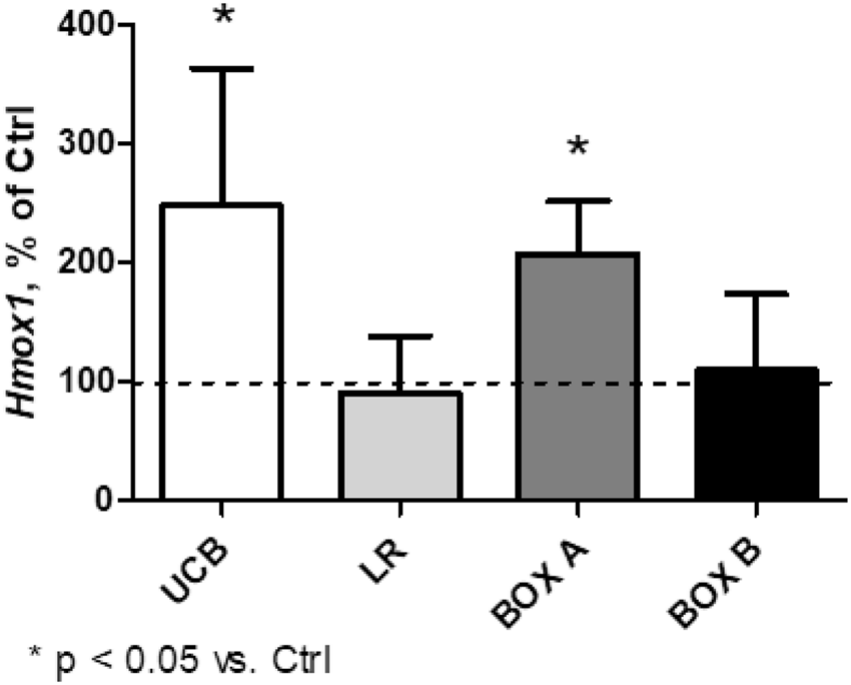
Expression of *Hmox1* mRNA in organotypic rat hippocampal slices after 4 h exposure to UCB, LR, BOX A, or BOX B. Expression of *Hmox1* in control organotypic rat hippocampal slices was set to 100%. Concentrations of tested compounds = 25 μmol/L.

The expression of heme oxygenase-1 (*HMOX1*), a marker of oxidative stress^23^ and the key enzyme in the heme catabolic pathway, was not affected in any of human CNS cell lines (data not shown) following a 4-h exposure to any of the studied compounds. In contrast, *HMOX1* expression was significantly upregulated in organotypic rat hippocampal slices incubated for 4 h with UCB or BOX A (2.5- and 2-fold, respectively, p < 0.05), but not with LR or BOX B (Fig. 3).

### The effect of bilirubin photo-oxidative products on the expression of pro-inflammatory genes in CNS models

Since UCB was demonstrated to have pro-inflammatory effects on neurons^24^, astrocytes^25^, and microglia^26^, we then analyzed the expression levels of the major pro-inflammatory genes.

**Figure 4:**
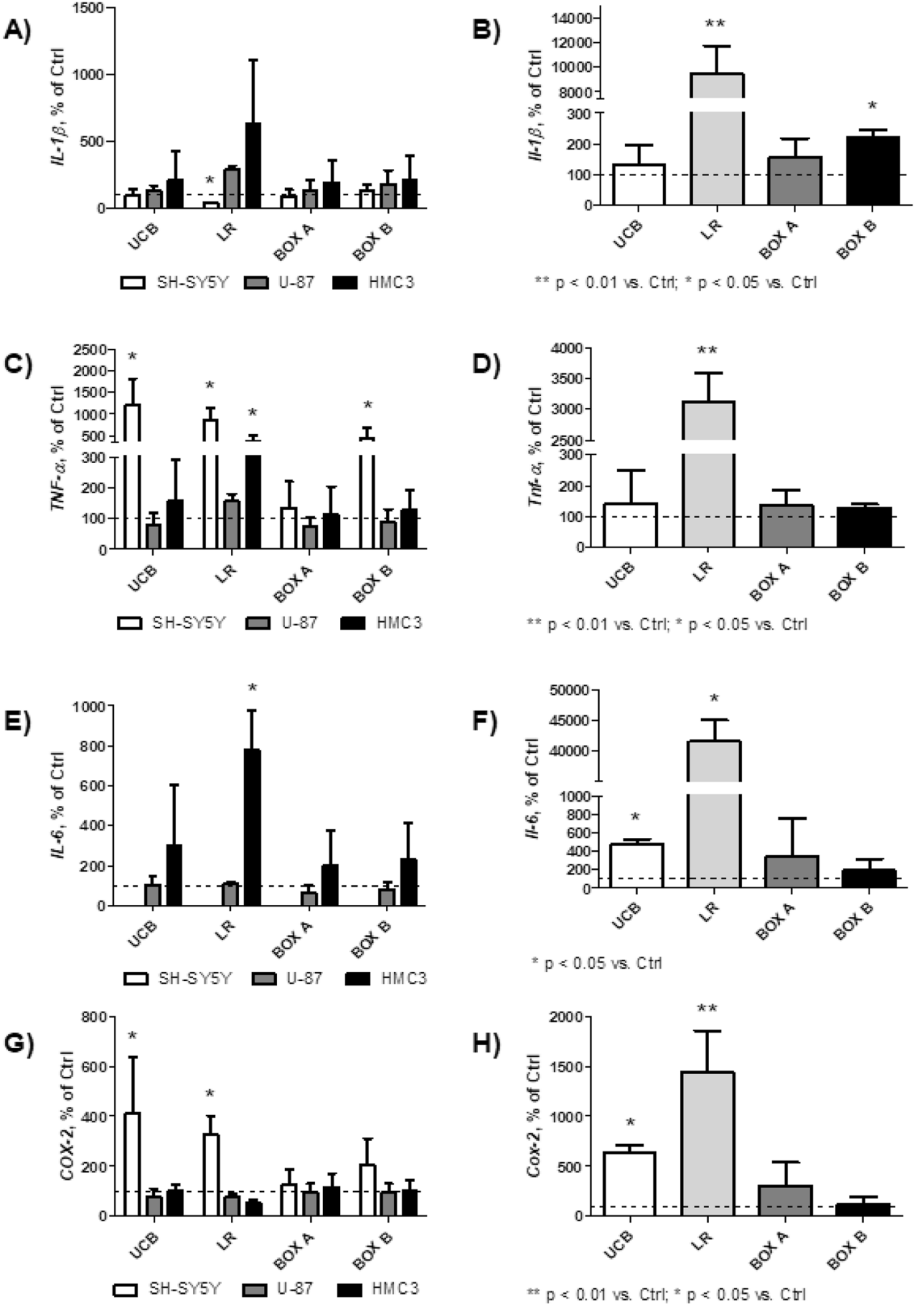
mRNA expressions of pro-inflammatory genes in CNS cells (**A**,**C**,**E**,**G**) and organotypic rat hippocampal slices (**B**,**D**,**F**,**H**). Four h exposure to UCB, LR, BOX A, or BOX B (25 μmol/L). Controls expressed as 100%. SH-SY5Y, human neuroblastoma cell line; U-87, human glioblastoma cells; HMC3, human microglia.

LR consistently increased the expression of all studied pro-inflammatory genes, TNF-α, IL-1β, IL-6, and cyclooxygenase-2 (COX-2), while the effects of other bile pigments were either negligible (BOX A) or mild (BOX B) (Fig. 4). Differences were apparent in all CNS cell lines, but were greater in organotypic rat hippocampal slices, with highly significant changes in the expression of all studied pro-inflammatory genes after LR exposure (Fig. 4). Increase in pro-inflammatory gene expression was observed for UCB (mostly on SH-SY5Y cells and organotypic rat hippocampal slices) (Fig. 4), but dramatic changes were observed only after LR exposure to all cell lines and organotypic rat hippocampal slices. In fact, the expression of TNF-α, IL-1β, IL-6, and COX-2 in organotypic rat hippocampal slices exposed to LR increased from 15- (COX-2) to 400-fold (IL-6) (Fig. 4).

### The effect of bilirubin photo-oxidative products on the production of TNF-α in organotypic rat hippocampal slices

**Figure 5:**
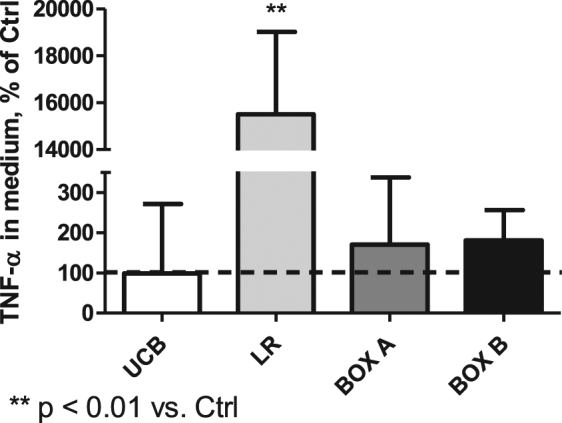
The effect of LR and other bilirubin photo-oxidative products on TNF-α production by organotypic rat hippocampal slices. Four h exposure to UCB, LR, BOX A, or BOX B (25 μmol/L). Controls expressed as 100%.

To confirm that overexpression of pro-inflammatory genes was mediated at the translational level, we evaluated the production of TNF-α in organotypic rat hippocampal slices. Although UCB had virtually no effect, BOX A and BOX B slightly (albeit not significantly) increased TNF-α concentration in the media. However, LR exposure led to a dramatic increase in TNF-α production (2423 vs. 16 ng/L, p < 0.01, Fig. 5).

## Discussion

Although millions of infants have been treated with PT for over the last five decades, recent clinical data suggest that PT might not be entirely harmless^4–11^, particularly in ELBW neonates, in whom increased morbidity as well as mortality has recently been reported. However, mechanistic studies on this subject are limited.

Here, we report that the major bilirubin photo-oxidative products (LR, BOX A, and BOX B) do not affect cell viability in any of the studied lines as well as in slices of rat hippocampus, the most vulnerable region affected in severe hyperbilirubinemia^22^.

In these studies, we focused mainly on the early phase of PT represented by a 4 h exposure of cells and tissues to bilirubin photodegradative products. In this early time period, plasma UCB concentrations are high and begin to decrease as LR concentrations, initially almost non-existent, start to increase. It can be inferred that LR concentrations vary between 25 to 50 µmol/L, with a decrease in plasma UCB concentrations to 30% during PT, and that no more than 20% of bilirubin PIs are *Z,E*-bilirubin^27^.

During this short-term exposure, neither LR nor bilirubin oxidative products (BOX A and B) caused any decreases in cell viability, even in G6PD-deficient fibroblasts, which have been shown to be very sensitive to oxidative stress. The gene expression of *HMOX1/Hmox1*, an important oxidative stress-response gene, was also only negligibly upregulated after exposure to LR, BOX A, or BOX B. Based on these observations, we concluded that bilirubin photo-oxidative products in biologically-relevant concentrations (25 µmol/L) present at the early stage of PT have no major effects on both cell viability and on the induction of oxidative stress. This is an important observation not only for LR, but also for BOX A and BOX B, which have been recently proposed to have an important pathophysiological role^18^. Moreover, our findings are consistent with the early studies by Silberberg *et al*. who did not detect any toxic effects of photo-irradiated bilirubin on myelinating cerebellum cultures^28^.

In contrast to our viability findings, we found a significant effect of LR on the expression of the major pro-inflammatory genes. It is noteworthy that PT given to hyperbilirubinemic neonates has been shown to be associated with possible lymphocyte genotoxicity^29^, increased concentrations of eosinophilic cationic protein^30^, as well as with an overall risk of childhood allergic diseases^5^. It is still not known whether these observations are due to a direct effect of PT light or to bilirubin photoderivatives generated during PT. Bilirubin *per se* is a well-recognized immunosuppressant^31^, although UCB has also been reported to induce, under specific conditions, the production of pro-inflammatory cytokines in astrocytes as well as in microglia^26,32^. However, no data has been reported that shows any immunomodulatory effects of bilirubin photo-oxidative products.

In our studies, we observed that after a short-term exposure to LR, there is a striking upregulation of pro-inflammatory genes (TNF-α, IL-1β, and IL-6), which encode major inflammatory cytokines that play crucial and multiple roles in the CNS^33^, including neurodevelopment with possible clinical implications such as impairment of neurogenesis^34^, development of childhood-onset mood disorders^35^, or major depressive disorders^36^. In fact, pro-inflammatory cytokine levels have been reported to increase over 100-fold over normal under pathological brain conditions – and indeed, we also show the same increase in TNF-α levels in media of organotypic rat hippocampal slices exposed to LR. Numerous studies have also reported that pro-inflammatory cytokines exert harmful effects on neurogenesis^34^. Upregulation of pro-inflammatory IL-1β, IL-6, and TNF-α triggers additional pathways, such as COX-2^37^, which is also implicated neuro-inflammation and neurogenesis^38^, although COX-2 seems to have an opposite effect compared to pro-inflammatory cytokines. The upregulation of COX-2 observed in our study might represent a feedback mechanism that is invoked to compensate for impaired (e.g. neurogenetic) signaling pathways^38^.

To further elucidate the specific mechanistic pathways involved in neuro-inflammation and exposure to photodegradative products of bilirubin, a more detailed investigation into the impact of PT on the inflammasome should be performed, and should also include *in vivo* studies to verify the clinical relevance of our data.

In conclusion, our data in context with reported roles of inflammatory cytokines collectively point to a possible explanation of the recently-reported potentially harmful effects of PT in small premature neonates^4–11^. In fact, LR seems to be more pro-inflammatory than the neurotoxic concentrations of unbound UCB (140 nM). Importantly, our results agree with the clinical data by Kurt *et al*.^39^, who reported an increase in systemic concentrations of circulating pro-inflammatory cytokines in hyperbilirubinemic newborn infants treated with PT, suggesting the translational relevance of our findings. Thus, the use of specific anti-inflammatory regimens initiated during intensive PT should be considered as a viable approach to reducing the adverse effects of PT in ELBW neonates.

## Methods

### Chemicals

Bilirubin (Sigma-Aldrich, St. Louis, MO, USA) was purified before use according to McDonagh and Assisi^40^. Methanol was purchased from Merck (Darmstadt, Germany), and ammonium acetate (NH_4_OAc) from Penta (Czech Republic). All other chemicals were purchased from Sigma.

### Preparation of bilirubin PIs

Pure bilirubin photo-derivatives were prepared as previously described^41^. Briefly, UCB (24 mg) was dissolved in 1 mL of 0.1 M NaOH and mixed with 1.2 g of rabbit serum albumin in PBS to a total volume of 50 mL. The solution was transferred to glass non-heparinized capillary tubes and exposed to 30 min of photo-irradiation using a Lilly photo-therapeutic device (TSE, Czech Republic), composed of an array of light-emitting diodes (LEDs) with a peak of 464 nm and wavelength range of 430–500 nm with an irradiance of 70 μW/cm^2^/nm corresponding to the total intensity of 2.2 mW/cm^2^. Each 5 mL of photo-irradiated sample was then precipitated with 25 mL of 0.1 M NH_4_OAc in CH_3_OH and centrifuged. The supernatant was evaporated under vacuum and the residue was dissolved in the mixture CHCl_3_:CH_3_OH (9:1, v/v) with a trace of TFA (20 µL) and then evaporated under vacuum again. Because of the light sensitivity of UCB and its PIs, all procedures were carried out under dim light and in aluminium-wrapped flasks. Evaporation was performed under vacuum and a stream of nitrogen.

### Thin layer chromatography

The residue after the latter evaporation was dissolved in a small amount of CHCl_3_:CH_3_OH:TFA mixture, and separated by thin layer chromatography (TLC) [200 × 200 × 0.25 mm Kieselgel 60 TLC plates (Merck, Darmstadt, Germany); mobile phase = chloroform:methanol:water, 40:9:1, v/v/v]. The upper yellow band was the residual bilirubin, the second yellow band was LR as verified by LC/MS^17^. LR was scraped out from the TLC plate, extracted with pure CH_3_OH from silicagel, and evaporated under a stream of N_2_. Evaporated LR was stored at −20 °C until use.

### Preparation of BOXes

The total synthesis of (Z)-2-(3-ethenyl-4-methyl-5-oxo-1,5-dihydro-2H-pyrrol-2-lidene) ethanamide (*Z*-BOX A) and (Z)-2-(4-ethenyl-3-methyl-5-oxo-1,5-dihydro-2H-pyrrol-2-ylidene) ethanamide (Z-BOX B) was prepared performed using a method reported previously^42^. TLC was performed on silica gel sheets (Merck TLC aluminium sheets silica gel 60 F254) and column chromatography on silica gel (60–230 mesh, Merck). NMR spectra were recorded on a VNMRS300, VarianUNITY *INOVA* 400 or Bruker Avance III 600 spectrometers, using 5-mm sample tubes. Internal references for ^1^H NMR: CDCl_3_: TMS (δ = 0.00); D_2_O: *t*-BuOH (δ = 1.25); DMSO: DMSO (δ = 2.50); ^13^C NMR: CDCl_3_: CDCl_3_ (δ = 77.0); D_2_O: *t*-BuOH (δ = 32.8); DMSO: DMSO (δ = 39.52). LR-MS (ESI) spectra were recorded on a Bruker Esquire 3000 with an ion-trap detector in positive or negative modes.

Purification by column chromatography (silica gel; chloroform/methanol 1:5, v/v) and crystallization from methanol yielded BOX A and BOX B, respectively, with spectral data were in accordance to those previously reported^42^.

### Cell line studies

*In vitro* studies were performed on three different human neuronal cell types (SH-SY5Y, U-87, and HMC3, ATCC, Manassas, VA, USA) and two types of fibroblasts [human dermal fibroblast cell line (No. C00425PA, Thermo Fisher Scientific, NJ, USA); and G6PD-deficient fibroblast cell line (GM01163 from 15-yr-old male) from Coriell Institute for Medical Research, Camden, NJ, USA]. Human SH-SY5Y cells were cultured in a mixture of MEM Eagle and Ham’s F12 media (1:1, v/v), containing 15% fetal bovine serum (FBS); human U-87 cells were cultured in DMEM containing 10% FBS, human HMC3 cells were maintained in MEM with 10% FBS; both types of fibroblasts were cultured in MEM with 15% FBS. All lines were maintained at 37 °C, in a 5% CO_2_ atmosphere. For functional tests, cells were seeded at a concentration of 50,000 cells/cm^2^. Authentication of cell lines was confirmed by an independent laboratory (Generi Biotech, Czech Republic).

### Organotypic rat hippocampal slice preparation

Wistar Han™ rats, at 8 (P8) days after birth (P: postnatal age in days), were obtained from the animal facility of the University of Trieste (Italy). Animal experiments were performed according to the Italian Law (decree 87–848) and European Community directive (86-606-ECC). The study was approved by the Animal Care and Use Committee of the University of Trieste along with regular communication with the competent Italian Ministry. The maximal effort was used to minimize the number of the animals used and their sufferance was done, in the respect of the 3R rule.

Organotypic rat hippocampal slices were prepared as previously described^22^. Immediately after sacrifice by decapitation, the hippocampus was harvested and maintained in dissection medium (ice cold Gey’s Balanced Salt Solution plus D-Glucose 10 mg/mL) until use. A McIIwain tissue chopper (Gomshall Surrey, UK) was used to cut transverse 350-μm slices. Healthy slices, selected for structural integrity under stereomicroscope inspection, were maintained in dissection medium for 60 min to allow clearing of cutting surfaces from preparation procedure stress. Slices were then transferred to a sterile, semi-porous Millicell-CM inserts (PICM03050, Millipore, Darmstadt, Germany), grown in 1 mL of medium, and maintained at 37 °C, 5% CO_2_, 95% humidity in a humidified incubator. The medium was changed the day after cutting and every two days thereafter. Slices were maintained in culture 5 days before starting treatment.

### Treatment

Fibroblasts were treated for 4 h with BOX A or BOX B in concentrations of 10, 50, 100, and 200 μmol/L containing DMSO corresponding to its concentration used for dissolving BOXes. SH-SY5Y, U-87, and HMC3 cells were treated with UCB, LR, BOX A, or BOX B in concentrations of 25 μmol/L for 4 h for both, viability and gene expression studies. Organotypic rat hippocampal slices were treated for 4 h with 25 μmol/L of UCB (corresponding to toxic concentration of 140 nmol/L of free bilirubin, Bf, based on our previous *in vitro* studies^43–46^), LR, and BOX A or BOX B starting after 5 days. The concentration of UCB dissolved in DMSO required to reach the desired Bf in the medium was quantified according to Roca *et al*.^47^. Control organotypic rat hippocampal slices were exposed to the same final concentration of DMSO used to dissolve the UCB.

### Cell viability analyses

The viability of fibroblasts was measured using a Cell-Counting Kit-8 (CCK-8, Dojindo Laboratories, MD, USA), utilizing WST-8 [2-(2- methoxy-4-nitrophenyl)-3-(4-nitrophenyl)-5-(2,4-disulfophenyl)-2H-tetrazolium salt], according to manufacturer’s instructions. After 4 h of incubation with CCK-8 solution, the absorbance was read at 450 nm. Results were expressed as percentage compared to the control cells (100%).

#### Mitochondrial activity

Mitochondrial metabolic activity was assessed using a 1-(4, 5-dimethyltiazol-2-yl)-3, 5-diphenylformazan (MTT) assay (Sigma-Aldrich). For post-challenge experiments, slices for each biological repetition were incubated with 0.5 mg/mL of MTT in medium at 37 °C for 1 h, harvested, and the precipitated salt dissolved in DMSO. Absorbance was detected at 562 nm using a LD 400 C luminescence detector (Beckman coulter, Milan, Italy). Results were expressed as percentage of activity related to the control (100%).

#### Lactate dehydrogenase release

The amount of total extracellular LDH in medium, indicative of membrane leakage, was determined using a CytoTox-ONE™ Homogeneous Membrane Integrity Assay (G7891, Promega, Madison, WI, USA). After treatment, the supernatant was collected and the reaction was carried out according to manufacturer’s instructions. The fluorescence (560 Ex/590 Em) was determined using an EnSpire Multimode Plate Reader (PerkinElmer, Waltham, MA, USA), and the background fluorescence subtracted. LDH in challenged slices was expressed as fold change compared to the control slices.

#### Hoechst staining

Evidence of chromatin condensation, a marker of cell death by apoptosis, was obtained by administration of 1 μg/mL Hoechst 33258 (Sigma-Aldrich). Slices challenged with UCB, LR, BOX A, BOX B, or DMSO alone were fixed for 30 min at room temperature in 3% paraformaldehyde. Apoptotic cells were counted at 40× magnification, by fluorescence microscopy using a Leica DM2000 (Leica Mycrosystems Srl, Solms, Germany) by three separate individuals. At least three different fields were analyzed in each biological repetition. Results were expressed as percentage of apoptotic cells related to the total number (apoptotic plus unaffected) cells in the control (100%).

#### Glutamate release

The concentration of extracellular glutamate, a marker of bilirubin-induced excitotoxicity, was quantified using a Glutamate Assay Kit (MAK004, Sigma-Aldrich). Briefly, after the treatment, the supernatant was collected, and the assay was performed according to manufacturer’s instructions. The absorbance (450 nm), proportional to the glutamate present, was determined using an EnSpire Multimode Plate Reader (PerkinElmer). Glutamate release in challenged-slice medium was expressed as fold change compared to the control slices.

### Quantitative real-time PCR

mRNA expression of genes of interest was analyzed by quantitative RT-PCR. Total RNA was extracted using TRI Reagent® RNA Isolation Reagent (Sigma-Aldrich) and GenUP Total RNA Kit (BiotechRabbit, Heningsdorf, Germany) from organotypic rat hippocampal slices or human cell lines, respectively, following the manufacturer’s instructions. Complementary DNA (cDNA) was synthesized with the High Capacity cDNA Reverse Transcription Kit (Applied Biosystems, Foster City, CA, USA). For the quantitative RT-PCR in organotypic rat hippocampal slices, primers were designed using the Beacon designer 4.2 software (Premier Biosoft International, Palo Alto, CA, USA) on rat sequences available in GenBank (Table 3). The reaction was performed in a final volume of 15 μL in an iQ5 Bio-Rad Thermal cycler (Bio-Rad Laboratories, Hercules, CA, USA). Briefly, 25 ng of cDNA and the corresponding gene-specific sense/antisense primers (250 nmol/L each, with the exception of *Cox2* and *Il1β*, 500 and 750 nM, respectively) were diluted in the Sso Advance SYBER green supermix (Bio-Rad Laboratories). Amplification of target genes was accomplished using the following protocol: 3 min at 95 °C, 40 cycles at 95 °C for 20 sec, 60 °C for 20 sec, and 72 °C for 30 sec. Specificity of the amplification was verified by a melting-curve analysis, and non-specific products of PCR were not found in any case. The relative quantification was made using the iCycleriQ software, version 3.1 (Bio-Rad Laboratories) by the ΔΔCt method, taking into account the efficiencies of the individual genes and normalizing the results to the housekeeping genes (*Hprt*, *Gapdh*)^48,49^.

**Table 3:** List of rodent genes used for gene expression analyses.

Gene	Forward	Reverse
*Hmox1*	GGTGATGGCCTCCTTGTA	ATAGACTGGGTTCTGCTTGT
*Il-1β*	AACAAGATAGAAGTCAAGA	ATGGTGAAGTCAACTATG
*Il-6*	TCCTTCCTACCCCAACTTCCAATG	CCACAGTGAGGAATGTCCACAAAC
*Tnf-α*	CAACTACGATGCTCAGAAACAC	AGACAGCCTGATCCACTCC
*Cox-2*	CTTTCAATGTGCAAGACC	TACTGTAGGGTTAATGTCATC
*Hprt1*	AGACTGAAGAGCTACTGTAATGAC	GGCTGTACTGCTTGACCAAG
*Gapdh*	CTCTCTGCTCCTCCCTGTTC	CACCGACCTTCACCATCTTG

*Hprt1* and *Gapdh* were used as the housekeeping genes.

RT-PCR of genes in human cell lines was performed on ViiA 7 instrument (Applied Biosystems) in 12-μL reaction volumes, containing 5 μL of 10-fold diluted cDNA template from a completed reverse transcription reaction, 1x SYBR Green Master Mix (Applied Biosystems), and 200–1000 nmol/L of forward and reverse primers. Data were normalized to *HPRT1* level and expressed in percentage to control. Primers of human genes used for RT-PCR analyses are given in Table 4.

**Table 4:** List of human genes used for gene expression analyses.

Gene	Forward	Reverse
*HMOX1*	atgccccaggatttgtca	cccttctgaaagttcctcat
*IL-1β*	acgatgcacctgtacgatca	ggaccagacatcaccaagct
*IL-6*	aggcactggcagaaaacaac	agctctggcttgttcctcac
*TNF-α*	acctcctctctgccatcaaga	ggaagacccctcccagataga
*COX-2*	ggtggagaagtgggttttca	acagcccttcacgttattgc
*HPRT1*	acatctggagtcctattgacatcg	ccgcccaaagggaactgatag

*HPRT1* was used as the housekeeping gene.

### Determination of TNF-α production

Determination of TNF-α concentrations in media of organotypic rat hippocampal slices exposed to UCB and bilirubin photo-oxidative products was measured using an ELISA test according to manufacturer’s instructions (ab100785, Abcam, Cambridge, UK). At the end of the treatments, cultures media were collected and stored at −80 °C until use. Briefly, media were incubated overnight at 4 °C degree in the plate coated with the primary antibody against TNF-α. Subsequently, the samples were incubated with biotinylated antibody and streptavidin-HRP solution. The reaction was started by adding the TMB solution for 30 minutes and then stopped by adding the Stop Solution. The absorbance (450 nm), proportional to the TNF-α present, was determined using an EnSpire Multimode Plate Reader (PerkinElmer). The range of TNF-α detection was 80–20,000 pg/mL, the sensitivity less than 25 pg/mL.

### Statistical analyses

Data are expressed as mean ± SD. Differences between variables were evaluated by the Mann-Whitney Rank Sum test. Differences were considered statistically significant at p < 0.05. Statistical analyses were performed using Prism 6 software (GraphPad, San Diego, CA, USA).
